# Navigation-Assisted Surgery for Locally Advanced Primary and Recurrent Rectal Cancer

**DOI:** 10.1245/s10434-023-13964-9

**Published:** 2023-07-23

**Authors:** Arne M. Solbakken, Simen Sellevold, Milan Spasojevic, Lars Julsrud, Hanne-Line Emblemsvåg, Henrik M. Reims, Olaf Sørensen, Ebbe B. Thorgersen, Lena Fauske, Joanna Sara Maria Ågren, Bjørn Brennhovd, Truls Ryder, Stein G. Larsen, Kjersti Flatmark

**Affiliations:** 1 Department of Gastroenterological Surgery, The Norwegian Radium Hospital, Oslo University Hospital, Oslo, Norway; 2 Institute of Clinical Medicine, University of Oslo, Oslo, Norway; 3 Department of Orthopaedic Oncology, The Norwegian Radium Hospital, Oslo University Hospital, Oslo, Norway; 4 Department of Radiology, The Norwegian Radium Hospital, Oslo University Hospital, Oslo, Norway; 5 Department of Pathology, Rikshospitalet, Oslo University Hospital, Oslo, Norway; 6 Department of Oncology, The Norwegian Radium Hospital, Oslo University Hospital, Oslo, Norway; 7 Department of Interdisciplinary Health Sciences, Institute of Health and Society, University of Oslo, Oslo, Norway; 8 Department of Urology, The Norwegian Radium Hospital, Oslo University Hospital, Oslo, Norway; 9 Department of Oncologic Plastic Surgery, The Norwegian Radium Hospital, Oslo University Hospital, Oslo, Norway; 10 Department of Tumour Biology, Institute for Cancer Research, The Norwegian Radium Hospital, Oslo University Hospital, Oslo, Norway

**Keywords:** Navigation-assisted surgery, Image-guided surgery, Optical tracking, Locally advanced rectal cancer, Locally recurrent rectal cancer, Feasibility study

## Abstract

**Background:**

In some surgical disciplines, navigation-assisted surgery has become standard of care, but in rectal cancer, indications for navigation and the utility of different technologies remain undetermined.

**Methods:**

The NAVI-LARRC prospective study (NCT 04512937; IDEAL Stage 2a) evaluated feasibility of navigation in patients with locally advanced primary (LARC) and recurrent rectal cancer (LRRC). Included patients had advanced tumours with high risk of incomplete (R1/R2) resection, and navigation was considered likely to improve the probability of complete resection (R0). Tumours were classified according to pelvic compartmental involvement, as suggested by the Royal Marsden group. The Brainlab^TM^ navigation platform was used for preoperative segmentation of tumour and pelvic anatomy, and for intraoperative navigation with optical tracking. R0 resection rates, surgeons’ experiences, and adherence to the preoperative resection plan were assessed.

**Results:**

Seventeen patients with tumours involving the posterior/lateral compartments underwent navigation-assisted procedures. Fifteen patients required abdominosacral resection, and 3 had resection of the sciatic nerve. R0 resection was obtained in 6/8 (75%) LARC and 6/9 (69%) LRRC cases. Preoperative segmentation was time-consuming (median 3.5 h), but intraoperative navigation was accurate. Surgeons reported navigation to be feasible, and adherence to the resection plan was satisfactory.

**Conclusions:**

Navigation-assisted surgery using optical tracking was feasible. The preoperative planning was time-consuming, but intraoperative navigation was accurate and resulted in acceptable R0 resection rates. Selected patients are likely to benefit from navigation-assisted surgery.

**Supplementary Information:**

The online version contains supplementary material available at 10.1245/s10434-023-13964-9.

## Introduction

Surgical treatment of patients with primary locally advanced rectal cancer (LARC) and locally recurrent rectal cancer (LRRC) involves multivisceral pelvic surgery beyond total mesorectal excision (TME).^1^ Since prognosis depends on resection margins, the aim is to remove the tumour with clear margins while preserving uninvolved structures important for physical functions.^2,3^ Surgery is often complex, with pelvic anatomy and extent of tumour growth varying from case to case, and fibrosis concealing anatomical landmarks, particularly in irradiated and recurrent tumours.^4^

In surgical disciplines where anatomy is complex and surgical accuracy critical, such as head and neck, orthopaedic and neurosurgery, navigation-assisted surgery has been implemented with success.^5–7^ In these settings, resections are performed in tissues where the displacement of the target structures is minimal as the dissection proceeds. The accuracy of navigation can therefore be maintained throughout the procedures. Since the rectum is encased by the pelvic bone with similar limited possibility of displacement of anatomical structures, navigation-assisted surgery could also be relevant for rectal cancer. A few case reports have examined navigation for rectal tumours requiring sacral resection and trans-anal TME,^8,9^ and in a recent study of LARC and LRRC, navigation-assisted surgery resulted in higher complete resection rates for LRRC cases compared with a historical non-navigated cohort.^10^ However, it is still unclear which navigation technology is best suited for implementation in rectal cancer surgery, and which patients will benefit the most.

With this in mind, we conducted the present study using navigation technology based on optical tracking, to evaluate the feasibility of navigation-assisted surgery for LARC and LRRC tumours requiring beyond-TME surgery.

## Materials and Methods

### Study Design and Patient Selection

The NAVI-LARRC (Computer navigation-assisted surgery for locally advanced and recurrent rectal cancer) trial was a prospective single-arm trial where the aim was to determine the feasibility of navigation-assisted surgery for LARC and LRRC (NCT 04512937). Surgery was planned and performed using the Brainlab^TM^ navigation system with Elements software and the Kick® Navigation Station (Brainlab, Munich, Germany) with navigation based on optical tracking with infrared light. Feasibility of navigation was assessed through a tripartite primary endpoint: the R0 resection rate, assessment of surgeons’ opinions on the navigation procedure through a study-specific questionnaire and individual interviews, and determination of adherence to the preoperative resection plan during surgery using postoperative magnetic resonance imaging (MRI). Study approval was obtained from the Regional Ethics Committee of South-East Norway (Approval ID 123753), and written informed consent was obtained from all patients.

All patients were treated at the Norwegian Radium Hospital, a tertiary referral centre for LARC and LRRC requiring surgery beyond TME, and part of Oslo University Hospital Comprehensive Cancer Centre. The study team consisted of two rectal cancer MRI radiologists, five colorectal and three orthopaedic surgeons performing resections, plastic and urologic surgeons providing reconstructive surgery according to need, and two pathologists with experience in evaluation of multivisceral specimens. Routine staging of patients included clinical examination under anaesthesia, computer tomography (CT) of the thorax, abdomen and pelvis, and pelvic MRI to determine tumour stage for LARC,^11^ and the location, size and number of lesions in LRRC cases. Inclusion criteria were LARC and LRRC with high risk of R1/R2 resection, where the multidisciplinary team (MDT) deemed that navigation-assisted surgery would be likely to improve the probability of obtaining R0 resection. Tumours were additionally classified according to the MRI compartmental classification of rectal cancer suggested by the Royal Marsden group, with involvement defined as tumour extending to (≤ 1 mm), or infiltrating structures.^12^ Thirty-day complications were classified according to the Accordion classification system.^13^ The first follow-up at 3 months consisted of clinical evaluation, a CT of the thorax, abdomen and pelvis, and the study-specific pelvic MRI to determine whether the resection plan had been followed.

### Preoperative Planning

**Fig. 1:**
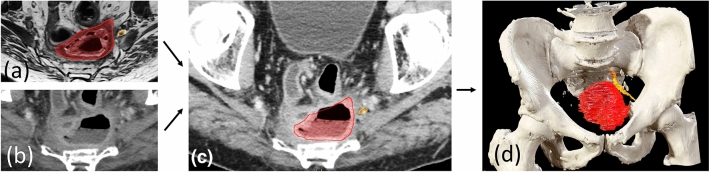
Preoperative segmentation and virtual 3-dimensional (3D) model. **a** Tumour (*red*) and the adjacent S2 nerve (*yellow*) manually segmented on axial MRI. **b** Axial CT image. **c** Segmented structures transposed to axial CT image after fusion of MRI and CT. **d** Virtual 3D model with automatically segmented pelvic bone and manually segmented tumour and S2 nerve

Preoperative planning was based on 2-dimensional (2D) MRI and a 3-dimensional (3D) virtual CT-derived model, showing the spatial relations between tumour and adjacent structures (Fig. 1 and Supplementary Video 1). To create the 3D model, the study radiologists first outlined tumour boundaries on 2D MRI (1.5 or 3 Tesla, 1-mm axial T2 weighted high-resolution slices) taken after neoadjuvant treatment. The MRI images were subsequently imported into the Brainlab^TM^ Elements platform where segmentation, i.e. delineation of tumour boundaries and key adjacent anatomical structures, was performed by a surgeon (AMS), in accordance with the radiologists’ assessment. MRI was thereafter fused with CT (soft tissue kernel, slice thickness ≤ 1 mm), whereby segmented anatomy was transposed to axial, sagittal and coronal CT images, and to a 3D model of the pelvic bone automatically segmented from the CT. Prior to surgery, the entire surgical team evaluated this model together with the conventional 2D MRI images, reaching consensus on a resection plan for each patient.

### Image-to-Patient Registration and Intraoperative Navigation

**Fig. 2:**
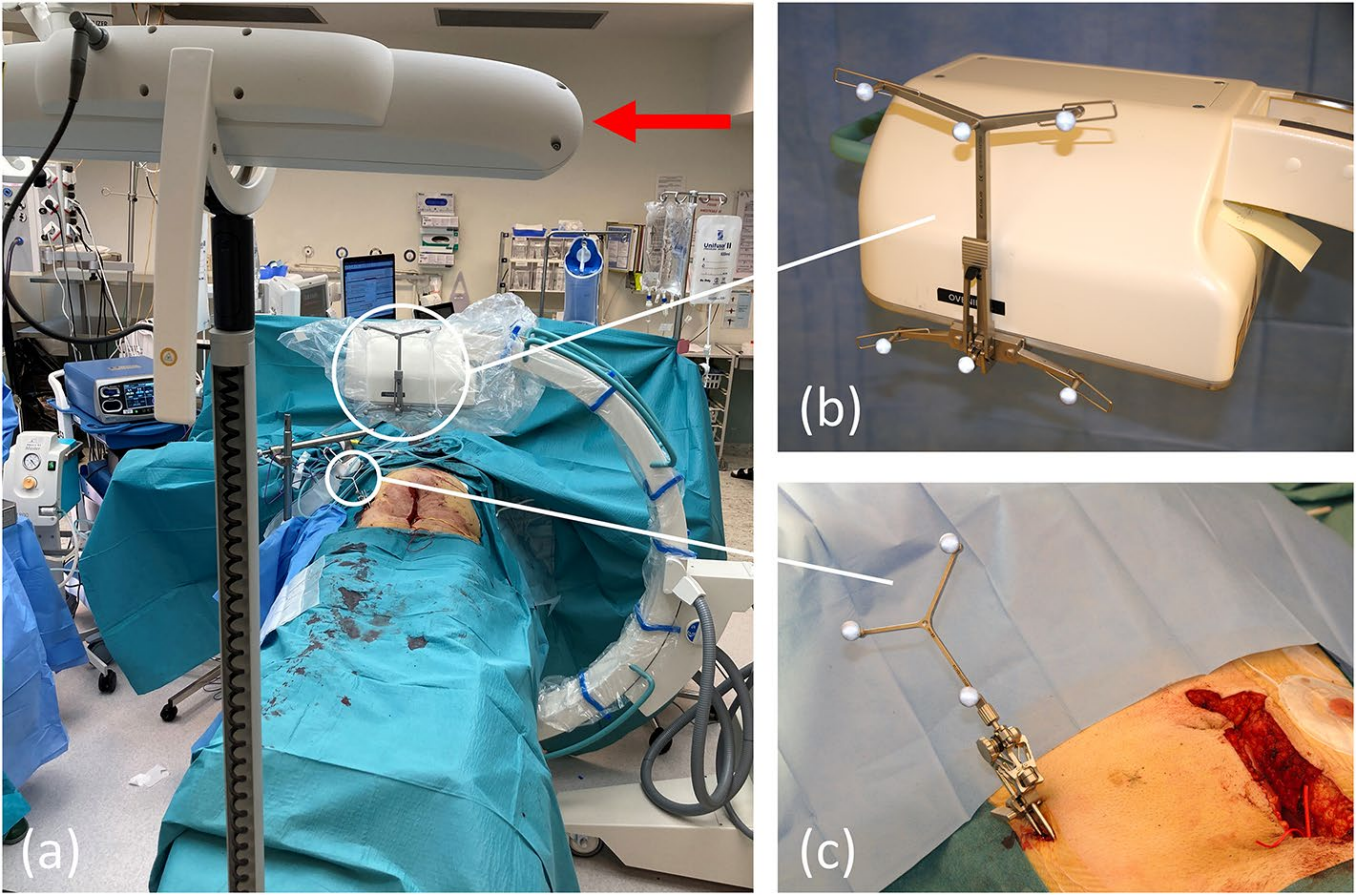
Intraoperative imaging. **a** Camera (*red arrow*) for optical tracking (with infrared light) of reflective spheres, on the fluoroscopy unit **b** and the patient tracker **c**. The position of fluoroscopy image data was registered relative to the patient tracker during image acquisition

**Fig. 3:**
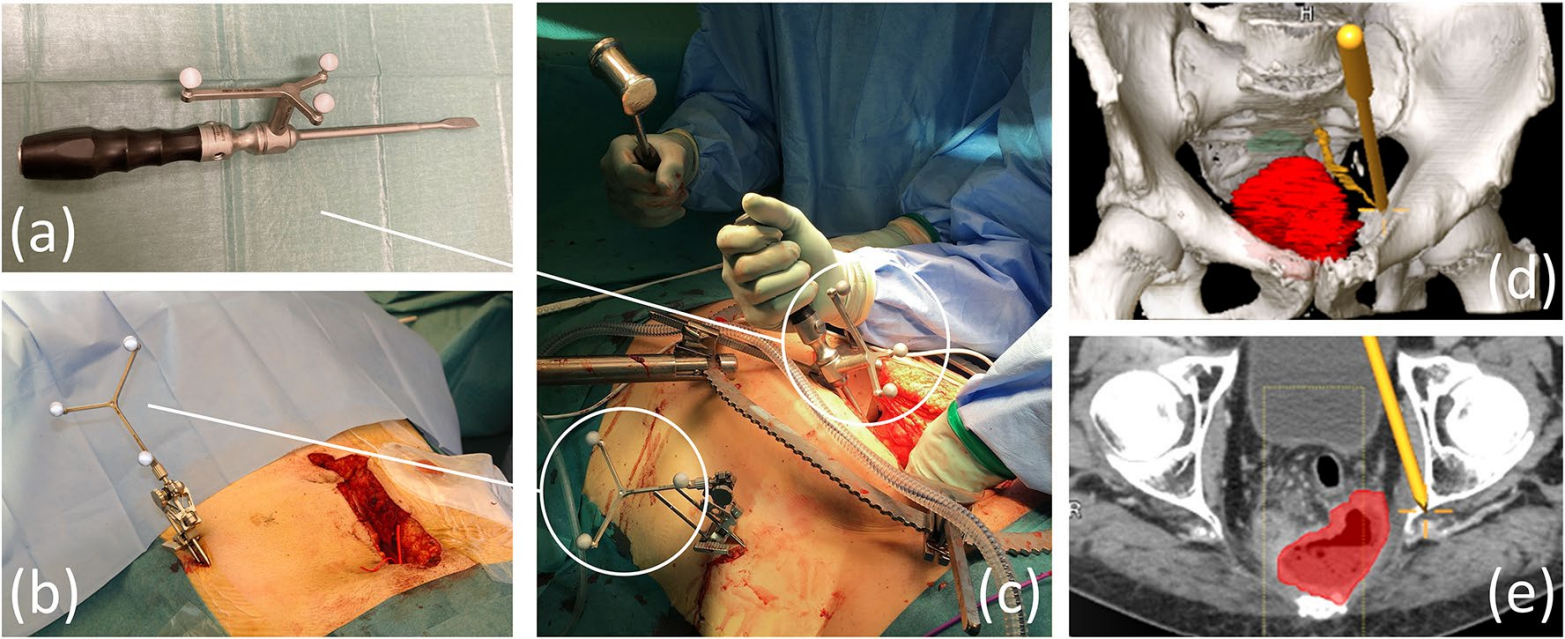
Intraoperative navigation. **a** Reflective spheres on chisel. **b** Reflective spheres on patient tracker. **c** Resection of the sciatic spine using a navigated chisel. **d** The chisel visualised in the 3D model. **e** The chisel visualised in the axial CT image containing the tip of the chisel

Intraoperative navigation was performed by visualising the surgical instruments in real-time on the preoperative CT images and in the 3D model. To achieve this, registration was performed, where the preoperative images were matched to the patient at surgery (Fig. 2 and Supplementary Video 2). With the patient in the supine position, a patient tracker was first fixed to the anterior iliac crest with two 5-mm threaded pins, ensuring a stable position relative to the pelvic bone. Intraoperative imaging of the patient’s pelvis was thereafter done with a 3D C-arm fluoroscopy unit (Ziehm Vision FD Vario 3D, Ziehm Imaging^TM^, Nürnberg, Germany). During image acquisition, a camera emitting infrared light was used for optical tracking of reflective spheres on the patient tracker and the fluoroscopy C-arm, allowing image data of pelvic bone from fluoroscopy to be positioned relative to the patient tracker. 3D fluoroscopy images were converted to 2D fluoroscopy images and subsequently matched with the preoperative CT images in the axial, sagittal, and coronal plane, thereby positioning also the preoperative CT images and the 3D model relative to the patient tracker. Subsequent simultaneous optical tracking of the patient tracker and reflective spheres on the surgical instruments allowed the computer to show the exact position of surgical instruments in real-time on axial, sagittal and coronal CT images and in the 3D model (Fig. 3 and Supplementary Video 3). The accuracy of the registration was determined in the cranio-caudal, antero-posterior and medio-lateral planes by placing the tip of a navigated pointer on exposed bony landmarks (i.e. pubic symphysis, sacral promontory, and iliac crest) to measure the mismatch (in mm) between the actual position in the patient and the position on the navigation screen. All navigation procedures were performed with the patient in the supine position.

### Assessment of the Surgeons’ Experiences with Navigation

**Fig. 4:**
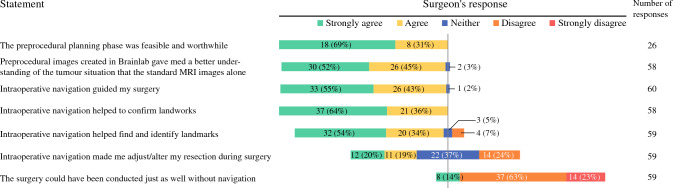
Surgeons’ responses to statements in questionnaire. The *numbers* indicate the number of a given response to a statement (percentages relative to the total number of responses to that statement)

After each procedure, participating surgeons completed the study-specific questionnaire containing seven statements with response options graded on a 5-point Likert scale (Fig. 4).^14^ The questionnaire focused on the value of segmentation and 3D imaging and explored the use and significance of navigation. The surgeons were also asked to specify structures that had been identified by navigation during surgery*.* After 12 procedures, a qualitative researcher (LF) conducted individual semi-structured interviews with the eight study surgeons, focusing on the intraoperative use of the navigation technology. Interviews were transcribed verbatim and analysed with a reflexive thematic approach, where inductive coding and division into categories and themes were done (by LF and AMS) to identify patterns of meaning.^15^ Written informed consent was given by all participating surgeons.

### Pathology

The surgical specimen was marked by the surgeon to identify anatomical structures and areas at risk of involved margins. In addition, the study pathologists were provided with a form indicating structures involved by tumour according to MRI. Histopathological examination was performed according to the recently published PelvEx Collaborative guidelines for pelvic exenterative practice.^16^ During macroscopic examination, the specimen slices were photographed and the images annotated for identification of specific areas of interest. Primary tumours were classified according to TNM 8.^11^ Response to neoadjuvant therapy was assessed using the four-tier system for tumour regression score (TRS) suggested by the College of American Pathologists.^17^ Circumferential resection margins (CRM) were classified as R0 (> 1 mm between the tumour and the resection margin), R1 (≤ 1 mm), or R2 (local macroscopic residual tumour) for both LARC and LRRC.^18^

### Post-hoc Evaluation of R1 Events

In patients with R1 resection, the specific location of margin involvement was annotated on photomicrographs, and on the macroscopic images of specimen slices. The corresponding preoperative MRI slice containing the segmented tumour was identified to visualise the structures involved in the R1 resections (Supplementary Fig. S1). This allowed assessment of whether the specific location was included in the original resection plan, and whether R1 resection occurred in navigated areas.

### Statistical Analyses

Since the study investigated feasibility, there was no formal inclusion target or power calculation. Results are presented as absolute numbers or median with min-max values unless otherwise stated. All data were prospectively registered.

## Results

### Patient Characteristics

Out of 226 patients with LARC and 19 with LRRC scheduled for beyond-TME surgery between 1 October 2020 and 1 October 2022, 20 patients were included in the study. Three patients were excluded upon MDT reassessment for the following reasons: navigation deemed unnecessary (*n* = 1), deferral from curative surgery due to age and comorbidity (*n* = 1), and irresectable metastatic disease (*n* = 1). The remaining 17 patients underwent navigation-assisted surgery with curative intent: LARC (*n* = 8) and LRRC (*n* = 9, including one patient with sigmoid cancer recurrence requiring sacral resection) (Table 1). All patients received neoadjuvant oncological treatment prior to surgery: chemoradiation (CRT) consisting of 2 Gy x 25 with concomitant capecitabine (*n* = 8), induction chemotherapy consisting of four cycles of chemotherapy followed by CRT (*n* = 3), re-irradiation with 1,2 Gy x 2 x 17 (*n* = 3) or 1,5 Gy x 2 x 15 (*n* = 2), and short-course radiation (5 Gy x 5) followed by consolidation chemotherapy (*n* = 1). All except one patient had ECOG performance status 0. The median number of compartments involved was 4 (3–5), with the lateral compartment being involved in all patients, and the posterior in 16 of 17 cases.

**Table 1:** Patients and tumour characteristics

Tumour	Patient number	Sex	Age	Neo-adjuvant therapy	ymriTNM	Compartments involved on MRI	Rectal resection	Sacral resection	CRM (mm)	Margin status
LARC	3	Male	74	CRT	T4bN1M0	Central, ABP, Posterior, Lateral, Infralevator	TPE	S4	6	R0
LARC	4	Male	57	ICT + CRT	T4bN1cM0	Central, ABP, Posterior ,Lateral, Infralevator	TPE	S4	6	R0
LARC	6	Male	73	CRT	T4bN0M0	Central, Posterior, Lateral, Infralevator	APR	S5	0	R1
LARC	8	Male	67	ICT + CRT	T4bN1cM1^a^	Central, Posterior, Lateral	TPE	S5	1,5	R0
LARC	10	Female	44	ICT + CRT	T4bN1cM0	Central, ABP, Posterior, Lateral, Infralevator	APR	–	3	R0
LARC	12	Male	61	CRT	T4bN0M0	Central, Posterior, Lateral, Infralevator	TPE	S3	5	R0
LARC	13	Female	74	CRT	T4bN0M0	Central, ABP, Lateral, Infralevator	APR	S5	0	R1
LARC	15	Male	50	CRT	T4bN0M0	Central, Posterior, Lateral	Hartmann	S4	5	R0
LRRC	1	Male	66	Re-irrad	–	Central, Posterior, Lateral, Infralevator	APR	S3	0	R1
LRRC	2	Male	59	CRT	–	Central, Posterior, Lateral	APR	S3	3	R0
LRRC	7	Male	69	Re-irrad	–	AAP, ABP, Posterior, Lateral	APR	S3	4	R0
LRCC	9	Male	68	CRT	–	Central, AAP, Posterior, Lateral	–	S1	0	R1
LRRC	14	Male	42	Re-irrad	–	AAP, Posterior, Lateral	APR	–	1,5	R0
LRRC	17	Female	62	CRT	–	Central, ABP, Posterior, Lateral, Infralevator	APR	S3	5	R0
LRRC	18	Male	57	Re-irrad	–	Central, ABP, Posterior, Lateral, Infralevator	TPE	S3	1	R1
LRRC	19	Female	49	Re-irrad	–	Central, AAP, Posterior, Lateral, Infralevator	APR	S2	2,5	R0
LRRC	20	Male	67	SCRT + CCT	–	Central, AAP, Posterior, Lateral, Infralevator	TPE	S3	30	R0

*LARC* locally advanced primary rectal cancer, *LRRC* locally recurrent rectal cancer, *LRCC* locally recurrent colon cancer, *CRT* chemoradiation, *ICT* induction chemotherapy, *Re-irrad* hyperfractionated re-irradiation, *SCRT+CCT* short course radiation with consolidation chemotherapy, *ymriTNM* TNM stage after neoadjuvant treatment, *AAP* anterior above peritoneal reflection, *ABP* anterior below peritoneal reflection, *TPE* total pelvic exenteration, *APR* abdominoperineal resection, *Hartmann* rectal resection with end colostomy, *Sacral resection* level of sacral resection, *CRM* circumferential resection margin.

^a^*M1*: Enlarged lymph nodes in the small bowel mesentery resected en bloc with a recurrent tumour

### Surgical Procedures and Short-term Outcome

Operations were performed a median time of 10 weeks (7–20 weeks) after neoadjuvant therapy, and a median 5 weeks (2–14 weeks) after the last MRI. Rectal resections consisted of total pelvic exenterations (TPE, *n* = 6), abdominoperineal resections (APR, *n* = 9), and rectal resection with end colostomy (Hartmann, *n* = 1). Fifteen patients had abdominosacral resections, the level of sacral resection being S3 or below, except in one patient who had high subcortical sacrectomy of S1–S3.^19^ Thirteen patients had resection of sacral nerves S3 or above, three with complete sciatic nerve resection (Table 2). Fifteen patients had perineal reconstruction with a pedicled vertical rectus abdominis myocutaneous flap. The median blood loss was 1.6 l (0.5–8.5 l) and 4.5 l (2.0–6.1 l), and median time of operation was 11 h (9.1–17.5 h) and 18 h (8.5–22.6 h) for LARC and LRRC, respectively.

**Table 2:** Segmented, navigated and resected structures

Compartment and structure	Number of patients		
	Segmented	Navigated	Resected
Tumour	17	1	17
*Anterior above peritoneal reflection*			
Ureter	2	0	3
Prostate/seminal vesicle	3	0	6
*Anterior below peritoneal reflection*			
Uterus/vagina	1	0	3
Small bowel	1	0	6
*Posterior compartment*			
Sacral neuroforamina	17	17	0
Lumbosacral nerves (L5-S3)	13	9	13
Sacrum	17	15	15
Gluteus maximus muscle	3	0	4
*Lateral compartment*			
Internal iliac vessels	16	0	13
Piriform muscle	5	0	12
Internal obturator muscle	4	0	8
Obturator nerve	2	0	0
Sacrospinal ligament/coccygeal muscle	10	3	14
Sciatic spine	17	16	5

The sacral nerve roots were transected at a more proximal level than planned in three patients: the left S1 nerve while controlling bleeding from the superior gluteal vessels (*n* = 1), the right S1 nerve during dissection of dense fibrotic presacral tissue (*n* = 1), and the right S2 nerve during resection of the sacrospinal ligament (*n* = 1). The first two instances occurred prior to initiating navigation, and the third after completing the navigation procedure.

Ten patients experienced Accordion complications grade 3 or above; mechanical ventilation more than 72 h (*n* = 1), deep surgical site infection requiring radiologic drainage (*n* = 8), and stenosis of the ureter requiring nephrostomy (*n* = 1). No patients required reoperation and there was no 30-day mortality, but one patient died 38 days after surgery with the autopsy showing acute pyelonephritis around an indwelling ureteral stent. The median time of hospital stay was 22 days (14–56 days).

### Segmentation and Registration

The navigation platform was used both in preoperative planning and for intraoperative navigation in all patients (Table 2). For preoperative planning, a median time of 3.5 h (1.5–6.3 h) were spent on segmentation, with a median 6 structures (5–11 structures) being segmented in each patient. At surgery, patient registration was completed in a median time of 42 min (30–73 min), and an accuracy of median 1 mm (0–3 mm) was achieved.

### Navigation Procedures According to the Surgeons

**Fig. 5:**
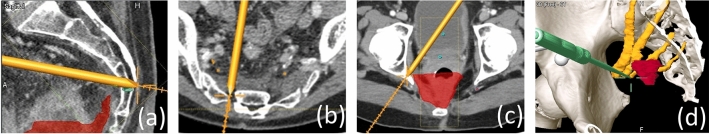
Structures identified and resected with navigation. **a** Sagittal CT image with navigated chisel resecting the sacrum S4, cranial to the tumour (*red*). **b** Axial CT image with navigated chisel identifying the right sacral neuroforamen S3. **c** Axial CT image with navigated chisel during resection of the right sciatic spine lateral to the tumour (*red*). **d** 3D model with navigated pointer (*green*) identifying the S2 nerve (*yellow*) distal to the tumour (*red*)

Eight participating surgeons completed a total of 60 study-specific questionnaires (Fig. 4). The surgeons agreed that the virtual 3D model with segmented structures gave a better understanding of the tumour situation than 2D MRI alone, and that navigation guided surgery by confirming or identifying landmarks. When asked whether the surgery could have been conducted just as well without navigation, most surgeons disagreed. Navigated structures were all located in the posterior and lateral compartments (Table 2), and most commonly included the neuroforamina with sacral nerves (*n* = 17), the level of sacral resection (*n* = 15), and the sciatic spine (*n* = 16) (Fig. 5).

In the individual interviews, intraoperative navigation was described as feasible and useful by all surgeons (Supplementary Table S1). Navigation provided increased confidence and resolve during dissection, and four out of eight surgeons thought they saved time by navigating. All surgeons considered that the inadvertent nerve resections could have been avoided with more extensive use of navigation. Seven of eight surgeons reported navigation to be particularly helpful in LRRC because of altered anatomy and fibrosis, resulting in more accurate tumour resection and better protection of vital structures compared with non-navigated surgery.

### Pathology

Intestinal adenocarcinoma was confirmed in all resected specimens, with moderate response to neoadjuvant treatment in most cases (TRS1, *n* = 2; TRS2, *n* = 14; TRS3, *n* = 1) (Supplementary Table S2). R0 resection was achieved in 6/8 LARC cases [CRM median 5 mm (1.5–6 mm)], and in 6/9 LRRC cases [CRM median 3.5 mm (1.5–30 mm)].

### Postoperative MRI

The 3-month postoperative MRI performed to evaluate adherence to the preoperative resection plan was available for 16 of the 17 patients (Supplementary Table S3). The postoperative MRI confirmed a high degree of adherence to the preoperative resection plan (165 out of 182 structures were resected as planned) with the following discrepancies: deliberate intraoperative adjustments (*n* = 8), MRI failing to identify partial resection of the pelvic sidewall fascia (*n* = 1) and pelvic muscles (*n* = 3), the inadvertent nerve resections (*n* = 3), and one patient where the planned resection of the obturator muscle (*n* = 1) and the sciatic spine was not performed (*n* = 1). Thus, the postoperative MRI provided little additional information relative to what was already recognised at the time of surgery.

### Evaluation of the R1 Events

Determining the location of R1 on preoperative MRI showed that these occurred in the vagina and paraprostatic tissue in the two LARC patients, in relation to the sacrospinal ligaments in two LRRC patients, and in fat surrounding a presacral tumour in the last LRRC patient (Supplementary Fig. S1). None of these structures had been segmented for navigation preoperatively, and in the last LRRC case, R1 occurred prior to initiating navigation.

## Discussion

The NAVI-LARRC inclusion criteria comprised any LARC or LRRC at risk of incomplete resection where navigation was likely to improve the probability for R0 resection, but only patients with tumours involving the posterior and lateral compartments were included after MDT discussion. In these compartments, surgery carries the highest risk of incomplete tumour resection and damage to vital structures.^20–22^ Also, tumours deep in these compartments lie in contact with the pelvic bone, ligaments and lumbosacral nerves. They will therefore retain their spatial position relative to the bone until resected, which permits preservation of navigation accuracy during dissection. Navigation was therefore expected to be of most benefit in these patients. In contrast, navigation was considered to be less useful in the other compartments where the potentially involved anatomical structures are mobile and at risk of being displaced from their original position relative to pelvic bone during surgery. Since the technology does not provide correction for such displacement, navigation could not be performed with similar accuracy for mobile structures. Accordingly, tumours located in the posterior and lateral compartment were deemed most relevant for navigation, with the sacrum with neuroforamina, the sacral nerves and the sciatic spine being the most commonly navigated anatomical landmarks.

The R0 rate in this study was 75% for LARC and 67% for LRRC cases. A study from the Netherlands Cancer Institute using navigation in 20 LRRC cases deemed to be at high risk of incomplete tumour resection recently reported a similar R0 rate of 70%.^23^ Another navigation study from the same institution reported an impressive R0 rate of 92.9% for LARC and 78.9% for LRRC, but the absence of details regarding compartments involved or structures resected makes it difficult to compare the complexity of surgical resections.^10^ In studies with LARC and LRRC where navigation was not used, but where patients required sacral or pelvic sidewall resections comparable to the present cohort, R0 rates of 62–82% were obtained, underlining the challenge of achieving clear margins in patients requiring extensive surgery in the lateral and posterior compartments.^20,21,24^ Given that the 17 NAVI-LARRC study patients represent the most advanced cases selected from 245 patients requiring beyond-TME surgery in the study period, we consider the R0 rates to be acceptable.

In four of the R1 events, the involved structures had not been segmented. The vagina and paraprostatic tissue were considered unsuitable for navigation due to potential displacement during dissection. The sacrospinal ligaments were not segmented because the resection plan involved navigation on the automatically segmented sciatic spine. In the last R1 case involving presacral fat, the segmented tumour could have been navigated, but R1 occurred before navigation was initiated. Consequently, involved structures were neither part of the 3D model used in preoperative planning, nor available for navigation at surgery, and the R1 resections do not therefore represent a failure of the navigation procedure per se. More extensive segmentation of tumour-adjacent structures could have increased the focus on the non-navigated tumour borders, and segmentation of the sacrospinal ligaments would have allowed navigation-assisted resection of these. Taken together, the R1 events therefore underline the importance of meticulous preoperative planning, supporting early and frequent use of navigation in such complex cases.

In the questionnaires and interviews, surgeons conveyed an essentially positive experience with the navigation platform. These results are in line with similar studies, where 3D models have been found useful for preoperative planning, and intraoperative navigation to guide surgery.^10,25,26^ A positive attitude caused by high expectations towards a novel, advanced technology could potentially have resulted in biased responses, overestimating the benefit of navigation in these studies. In the present study, the surgeons advocated extended use of navigation, which together with the uniform and consistent responses suggest that the high confidence in the navigation platform reflects the actual experience obtained through the trial.

Completion of preoperative segmentation and intraoperative registration using the Brainlab^TM^ platform required a median time of 3.5 h and 42 min, respectively, resulting in an accuracy of median 1 mm. The segmentation process seems equally time-consuming (1–3 h), independent of the software platform used,^27–29^ while the time needed for registration is more platform dependent. The two methods used for navigation in rectal cancer surgery are optical tracking using infrared light, as in this study,^30,31^ or electromagnetic tracking, where electromagnetic sensors attached to the patient and the surgical instruments are tracked in an electromagnetic field.^23,32^ While image-to-patient registration is more time-consuming for optical tracking than for electromagnetic tracking (25–47 min vs 16–21 min),^9,23,32,33^ studies using optical tracking tend to report superior accuracy (0.5–3.7 mm vs 3–4 mm).^9,10,29,34^ In the present study, registration times were in the upper range, but the resulting accuracy of median 1 mm was satisfactory.

Navigation limited to resection of bone can be performed merely based on CT images and automated segmentation of pelvic bone. However, further segmentation is required to take full advantage of the navigation platforms, with 3D models showing spatial relations and navigation of soft tissue structures for resection or preservation. In the future, dedicated imaging protocols, and computer-based image processing might provide automated segmentation of soft tissue, which would reduce the time spent on preoperative planning.^35–38^ At present, however, the time needed for segmentation represents the major obstacle for clinical implementation irrespective of the navigation platform, limiting navigation to selected patients. The small cohort of 17 patients makes it challenging to draw definitive conclusions regarding whether implementation of time-consuming navigation can be justified, particularly in experienced centers. Access to the navigation technology also represents an obstacle to more widespread use, both due to the cost of the equipment and the operational competence needed to use the navigation platform.^39,40^ However, for our group, navigation was viewed as helpful, and clear resection margins were obtained in the majority of patients despite very advanced tumours.

## Conclusion

Navigation-assisted surgery using optical tracking was feasible in advanced rectal cancer patients with tumours in the posterior and lateral compartment. Although time-consuming to generate, the 3D model of tumour and adjacent structures was helpful in preoperative planning, and intraoperative navigation was accurate, resulting in acceptable R0 resection rates. The involved costs, time requirements and complexity of the procedures currently limit this technology to dedicated centres, but selected patients are likely to benefit from navigation-assisted surgery.

### Supplementary Information

Below is the link to the electronic supplementary material.

Supplementary file1 (TIF 45483 KB)

Supplementary file2 (DOCX 19 KB)

Supplementary file3 (MP4 28437 KB)

Supplementary file4 (MP4 47854 KB)

Supplementary file5 (MP4 59090 KB)
