# Supplementary material for: Navigation-Assisted Surgery for Locally Advanced Primary and Recurrent Rectal Cancer
Source: Ann Surg Oncol. 2023 Jul 23;30(12):7602–11. doi: 10.1245/s10434-023-13964-9 (PMC10562504; doi:10.1245/s10434-023-13964-9)
Supplement: Supplementary file 2 — Supplementary file2 (DOCX 19 KB) [file 10434_2023_13964_MOESM2_ESM.docx]

**Suppl. Table S1** Selected quotations from interviews with surgeons.

| Theme | Quotation | Surgeon  number |
| --- | --- | --- |
| Feasibility of navigation-assisted surgery | Yes, [navigation was feasible both in preoperative planning and intraoperative navigation], because we get better precision, increased anatomical understanding, and better surgery in the operated patients.  Yes [intraoperative navigation] is absolutely feasible. There have been some challenges while operating in the [lithotomy] position etc., but we have overcome these, after exploring different approaches. So definitely feasible, with adaptations based on our experiences.  Yes, [intraoperative navigation was feasible] I think. It was useful [… and] clearly gave helpful additional information, an aspect which made us feel safer and better when operating the patients […] It is appropriate to do this for this group of patients. | 6  8  5 |
| Use of the navigation equipment | We use navigation in two settings: first, to identify landmarks, for orientation […] and second, to resect bone at the right place with a [navigated] chisel.  Concerning the sacrum and neuroforamina where the nerve roots come out: You remove various structures in front [of them …], and then you have a hypothesis – where are we now? – and you use the pointer to confirm, contradict or correct this with navigation.  [Navigation guided surgery] by identifying anatomical structures […], to protect structures we were to spare, and to resect those we were to include. | 2  4  6 |
| Benefits of navigation | [Intraoperative navigation] gives an increased confidence […] and calm in the surgical dissection.  The anatomy in these selected patients is challenging, and we were more sure on where to resect due to the navigation  Intraoperatively, I believe it has given increased confidence, and made it easier to continue the operation.  If you look at time needed to set up the navigation equipment – 42 minutes – that is quite a long time. But at the same time, you gain time – maybe even more sometimes – by operating more targeted and quicker, because you get a continuous confirmation that you are in the right place according to the preoperative plan. | 2  6  1  8 |
| Inadvertent nerve resections | I was involved in a patient where I divided the nerve root [inadvertently]. The problem was not navigation. If we had used navigation earlier there, we probably would have [avoided it].  The point with navigation is that you should use it before you arrive […]. We thought the operation progressed fine, and then we cut a sacral nerve root […] which would not have happened if we had stopped and used navigation at a more fixed time-point. If you wait until you need it, it might be too late.  It is important that if you have decided to use navigation in an operation, you should start navigation as early as possible to avoid such mistakes [the nerve damages]. | 7  3  8 |
| Recurrent rectal cancer | In recurrences where it is difficult to separate nerve roots from fibrosis there is a significant benefit with navigation, since you know where it is safe to go.  Fibrosis after radiation or previous surgery distorts anatomy to a great extent making it difficult to identify landmarks […] Then navigation is a very useful tool to help find where structures are located. | 1  8 |

**Suppl. Table S2**. Histopathology results

|  |  | Number of patients | |
| --- | --- | --- | --- |
|  |  | Primary rectal cancer | Recurrent colorectal cancer |
| Adenocarcinoma NOS |  | 6 | 9 |
| Mucinous adenocarcinoma |  | 2 | 0 |
| ypT-stage | ypT3 | 5 | - |
|  | ypT4b | 3 | - |
| ypN-stage | ypN0 | 4 | - |
|  | ypN1 | 0 | - |
|  | ypN1c | 3 | - |
|  | ypN2a | 1 | - |
| Unifocal recurrence | | | 9 |
| Tumour budding | Bd1 (low) | 7 | 5 |
|  | Bd2 (intermediate) | 0 | 2 |
|  | Bd3 (high) | 1 | 1 |
|  | Not determinable (autolysis) | - | 1 |
| Perineural invasion |  | 4 | 8 |
| Extramural vascular invasion |  | 1 | 1 |
| Tumour regression score | TRS 0 | 0 | 0 |
|  | TRS 1 | 1 | 1 |
|  | TRS 2 | 7 | 7 |
|  | TRS 3 | 0 | 1 |
| Margin status | R0 (> 1mm margin) | 6 | 6 |
|  | R1 (≤ 1 mm margin) | 2 | 3 |
| *NOS* not otherwise specified |  |  |  |

**Suppl. Table S3.** Comparison between resection plan and postoperative MRI (available for 16 out of 17 patients)

|  | Planned resected | Identified resected | Deviations from plan | Reason for discrepancy between resection plan  and resection performed according to MRI |
| --- | --- | --- | --- | --- |
| Rectum / Neo-rectum | 14 | 14 | 0 |  |
| Ureter | 3 | 4 | 1 | In patient 18, the planned APR was changed to TPE during surgery due to fibrosis. |
| Common iliac vessels | 0 | 0 | 0 |  |
| Urinary Bladder | 5 | 6 | 1 | In patient 18, the planned APR was changed to TPE during surgery due to fibrosis. |
| Prostate | 5 | 6 | 1 | In patient 18, the planned APR was changed to TPE during surgery due to fibrosis. |
| Seminal vesicle | 7 | 7 | 0 |  |
| Vagina | 2 | 2 | 0 |  |
| Uterus | 2 | 2 | 0 |  |
| Ovaries/Fallopian tubes | 1 | 1 | 0 |  |
| Presacral fascia | 16 | 16 | 0 |  |
| Sacrum - most proximal vertebrae |  |  |  |  |
| S1 sacrum | 1 | 1 | 0 |  |
| S2 sacrum | 1 | 1 | 0 |  |
| S3 sacrum | 7 | 7 | 0 |  |
| S4 sacrum | 4 | 3 | 1 | In patient 8, S5 was resected instead of S4 because the tumour was mobile at S4. |
| S5 sacrum | 2 | 3 | 1 | In patient 8, S5 was resected instead of S4 because the tumour was mobile at S4. |
| Sacral nerve - most proximal nerve |  |  |  |  |
| S1 sacral nerve | 2 | 4 | 2 | In patient 4 and 9, the S1 nerve was inadvertently resected. |
| S2 sacral nerve (right side) | 2 | 3 | 1 | In patient 19, the S2 nerve was inadvertently resected. |
| S2 sacral nerve (left side) | 3 | 3 | 0 |  |
| S3 sacral nerve | 5 | 5 | 0 |  |
| S4 sacral nerve | 2 | 1 | 1 | In patient 8, S5 was resected instead of S4 because the tumour was mobile at S4. |
| S5 sacral nerve | 1 | 2 | 1 | In patient 8, S5 was resected instead of S4 because the tumour was mobile at S4. |
| Sciatic nerve | 2 | 2 | 0 |  |
| Pelvic sidewall fascia | 14 | 13 | 1 | In patient 12, MRI failed to identify the actual removal of the pelvic sidewall fascia. |
| External iliac vessels | 0 | 0 | 0 |  |
| Internal iliac vessels | 12 | 12 | 0 |  |
| Piriformis muscle | 12 | 10 | 2 | In patient 12 and 14, MRI failed to identify partial resection of the piriform muscle. |
| Coccygeal muscle /sacrospinal ligament | 14 | 14 | 0 |  |
| Internal obturator muscle | 7 | 5 | 2 | In patient 1, MRI failed to identify partial resection of the internal obturator muscle. |
|  |  |  |  | In patient 6, resection was done medial to the planned resection of internal obturator muscle. |
| Sciatic spine | 7 | 6 | 1 | In patient 6, resection was done medial to the planned resection of sciatic spine. |
| Levator ani muscle | 13 | 13 | 0 |  |
| Anus / External sphincter complex | 13 | 13 | 0 |  |
| Ischioanal fossa / perineal scar | 3 | 3 | 0 |  |
| Urethra | 0 | 1 | 1 | In patient 18, the planned APR was changed to TPE during surgery due to fibrosis. |
| Crus penis / clitoris | 0 | 0 | 0 |  |
| *APR* abdominoperineal resection, *TPE t*otal pelvic exenteration | | | |  |
